# Supermeres Containing the Discoidin Domain Receptor 1 Extracellular Domain Promote Collagen Alignment and Immune Exclusion in Microsatellite-Stable Colorectal Cancer

**DOI:** 10.1158/2767-9764.CRC-26-0105

**Published:** 2026-09-14

**Authors:** Maxwell S. Hamilton, Samuel T. Ellis, Zheng Cao, Oleg S. Tutanov, Alan J. Simmons, Radhika Aramandla, James N. Higginbotham, Marisol Ramirez, Katherine W. Schnee, Matthew E. Bechard, Harsimran Kaur, Jeffrey L. Franklin, Swastika Mandal, Ken S. Lau, Qi Liu, Jeremy A. Goettel, Ambra Pozzi, Robert J. Coffey

**Affiliations:** 1 Program in Cancer Biology, Vanderbilt University, Nashville, Tennessee.; 2 Department of Medicine, Vanderbilt University Medical Center, Nashville, Tennessee.; 3 Epithelial Biology Center, Vanderbilt University Medical Center, Nashville, Tennessee.; 4 Department of Cell and Developmental Biology, Vanderbilt University School of Medicine, Nashville, Tennessee.; 5 Center for Computational Systems Biology, Vanderbilt University, Nashville, Tennessee.; 6 Department of Biostatistics, Vanderbilt University, Nashville, Tennessee.; 7 Program in Chemical and Physical Biology, Vanderbilt University, Nashville, Tennessee.; 8 Vanderbilt-Cancer Ingram Center, Vanderbilt University Medical Center, Nashville, Tennessee.; 9 Department of Pathology, Microbiology, and Immunology, Vanderbilt University Medical Center, Nashville, Tennessee.; 10 Division of Nephrology and Hypertension, Department of Medicine, Vanderbilt University Medical Center, Nashville, Tennessee.; 11 Department of Physiology and Molecular Biophysics, Vanderbilt University School of Medicine, Nashville, Tennessee.; 12 Department of Veterans Affairs Hospital, Tennessee Valley Healthcare System, Nashville, Tennessee.

## Abstract

Immune checkpoint blockade (ICB) is an effective treatment for microsatellite instability–high (MSI-H) colorectal cancers that are highly infiltrated by CD8^+^ T cells. Microsatellite-stable (MSS) colorectal cancers are unresponsive to ICB, at least in part, due to the paucity of intratumoral CD8^+^ T cells. We recently identified discoidin domain receptor 1 (*DDR1*) as one of four genes associated with CD8^+^ T-cell exclusion in MSS colorectal cancer. There are conflicting reports about the presence and role of the cleaved ectodomain (cECD) of *DDR1* in mouse models of breast and pancreatic cancers. To explore the role of the DDR1 cECD in human colorectal cancer, we developed Collagen Alignment and Spatial Transcriptomics Analysis (CASTA), which revealed that genes involved in fibroblast contractility were associated with both *DDR1* tumor expression and collagen alignment as determined by label-free second harmonic generation (2HG) imaging of the tumor collagen. Using 3D collagen cocultures of colorectal cancer spheroids and fibroblasts, we show that DDR1 promotes collagen alignment and CD8^+^ T-cell exclusion. We found large amounts of DDR1 cECD in MSS colorectal cancer supermeres, 25- to 35-nm secreted amembranous nanoparticles. Supermeres containing DDR1 cECD were sufficient to induce contraction of human colonic fibroblasts. We propose a model in which supermeres containing the DDR1 cECD promote the contraction of stromal fibroblasts in MSS colorectal cancer, leading to collagen alignment and CD8^+^ T-cell exclusion. These results support DDR1 cECD as an attractive therapeutic target in MSS colorectal cancer.

**Significance:**

In human colorectal cancer, supermeres containing the DDR1 ectodomain promote fibroblast contraction and collagen alignment to exclude intratumoral CD8^+^ T cells.

## Introduction

Immune checkpoint blockade (ICB) represents a major clinical advance in the treatment of colorectal cancer, in which it leads to durable remissions (1). However, the efficacy of ICB is limited to the 15% of patients with microsatellite instability–high (MSI-H) colorectal cancer that are characterized by intratumoral infiltration of cytotoxic CD8^+^ T cells (2). The remaining 85% of patients with microsatellite-stable (MSS) colorectal cancer do not respond to ICB (3). The increased CD8^+^ T-cell infiltration in MSI-H colorectal cancer is often attributed to increased mutational burden, resulting in increased neoantigen presentation (4). This model could be interpreted to mean that the use of ICB in MSS colorectal cancer is untenable, as MSS colorectal cancers have comparatively few mutations. However, recent work has challenged that assumption (4). We recently showed that increased CD8^+^ T-cell infiltration precedes hypermutation in MSI-H colorectal cancer precursor lesions, suggesting that hypermutation is dispensable for high CD8^+^ T-cell infiltration (2, 5). Moreover, tumors with low mutational burdens present neoantigens, for instance, by transcription of neo-open reading frames, resulting in the presentation of cryptic neoantigens (6, 7). As a result, an approach to increase the infiltration of CD8^+^ T cells into MSS colorectal cancer tumors has the potential to increase the number of patients eligible for ICB.

We recently described a four-gene signature that correlated with CD8^+^ T-cell exclusion in MSS colorectal cancer (8). One of the genes encodes discoidin domain receptor 1 (DDR1), a receptor tyrosine kinase (RTK) that is activated upon binding to fibrillar and nonfibrillar collagens (9, 10). This binding is reported to be coupled to proteolytic cleavage of DDR1 at its extracellular juxtamembrane region, leading to the release of the cleaved ectodomain (cECD) into the extracellular space (11–13).

DDR1 has been shown to be involved in immune regulation (14–20), as well as fibrosis in the colon (21), making it an attractive candidate for further study. In mouse breast cancer xenografts, DDR1 expression resulted in increased extracellular collagen fiber alignment, resulting in CD8^+^ T-cell exclusion (22, 23). DDR1 cECD was sufficient to cause exclusion in this model, indicating that DDR1 regulates collagen remodeling through a kinase-independent, non–cell-autonomous mechanism. Further work revealed that DDR1 was also cancer-promoting in pancreatic cancer. However, DDR1 expression was not associated with increased collagen alignment and the cECD was not detected, suggesting that DDR1 may promote cancer by different mechanisms in different cancer types (24). Although the role of DDR1 in CD8^+^ T-cell exclusion in colorectal cancer has been investigated, the studies have focused on full-length DDR1, have not explored the role of DDR1 cECD in collagen alignment, and have been limited to MSI-H mouse xenograft models (14). Therefore, we sought to explore the potential role of DDR1 cECD in promoting CD8^+^ T-cell exclusion in human colorectal cancer, focusing on its effects on collagen alignment.

Recombinant DDR1 ECD can promote collagen organization *in vitro* (25). However, how DDR1 cECD causes collagen alignment in a tumor context is poorly understood. Thus, the focus of this study was to better understand how DDR1 cECD promotes collagen alignment *in vivo* in colorectal cancer. We find that secreted DDR1 cECD is largely found in MSS colorectal cancer supermeres, 25- to 35-nm amembranous secreted nanoparticles we discovered several years ago (26). To investigate the genes, pathways, and cell types associated with collagen alignment in human colorectal cancer tumors, we developed Collagen Alignment and Spatial Transcriptomics Analysis (CASTA). Applying this method to tissue blocks of five MSS colorectal cancer tumors, we found that tumoral *DDR1* expression and collagen alignment were associated with increased expression of fibroblast genes involved in cell contractility in the stroma. We then developed a 3D colorectal cancer spheroid/fibroblast/T-cell coculture system which we use to show that supermeres containing DDR1 cECD released from colorectal cancer cells promote collagen alignment, fibroblast contractility, and CD8^+^, but not CD4^+^, T-cell exclusion. We propose a model in which DDR1 cECD is released from colorectal cancer cells in supermeres, resulting in increased contractility in fibroblasts, leading to collagen alignment and CD8^+^ T-cell exclusion.

## Materials and Methods

### Cells

DiFi cells are a female MSS colorectal cancer cell line developed in the Coffey laboratory (RRID: CVCL_6895; ref. 27). Cystic clone 3 (CC-3), spikey clone 1 (SC-1), and cetuximab-resistant cystic clone (CC-CR) cell lines are clones derived previously from HCA-7 cells obtained from ATCC (28, 29). Other colorectal cancer cell lines were obtained from ATCC. Fibroblast cell lines were previously isolated from the distal surgical resection margin of one patient with hereditary nonpolyposis colorectal cancer (denoted HNPCC) and one patient with sporadic colorectal cancer (denoted HCF; ref. 30). *Mycoplasma* testing was performed prior to use of the cells in experiments. Cell line authentication was performed by short tandem repeat profiling. Primary fibroblasts were isolated from the adjacent normal tissue of colorectal cancer and adenoma resections from consenting patients at the Vanderbilt University Medical Center [VUMC; institutional review board protocol (IRB) #151721] using a method adapted from Rockman and colleagues (31). Following isolation of colonic crypts, tissue was finely chopped and placed in complete medium as described in the “Two-dimensional cell culture” section with 15% bovine growth serum in a 10-cm dish. Once fibroblasts had adhered, remaining tissue was removed. Fibroblast isolation was performed in the presence of Primocin to prevent *Mycoplasma* contamination. Medium was changed every 3 to 4 days. CD4^+^ and CD8^+^ T cells were recovered from healthy donor peripheral blood mononuclear cells purchased from ATCC (cat. #PCS-800-012, lot 80204253). Cells were stained with FITC-conjugated anti–human CD3 (BioLegend, 344815), CD4 (BioLegend, 317407), and CD8 (BioLegend, 100733) antibodies in FACS buffer (PBS with 2% FBS and 2 mmol/L ethylenediaminetetraacetic acid) for 15 minutes at 4°C, washed in PBS, spun at 350 × *g* for 5 minutes, and finally resuspended in FACS buffer. CD4^+^ and CD8^+^ cells were sorted using a BD FACSMelody (BD Biosciences). Sorted CD8^+^ and CD4^+^ T cells were pelleted at 350 × *g* and resuspended in ImmunoCult Human CD3/CD28 T Cell Activator in ImmunoCult-XF T Cell Expansion Medium (STEMCELL Technologies) supplemented with recombinant human IL2 (Thermo Fisher Scientific).

### Two-dimensional cell culture

Colorectal cancer cell lines and fibroblasts were grown in DMEM (Corning, 10-017-CV) supplemented with 10% (colorectal cancer cell lines, HNPCC, and HCF) or 15% (primary fibroblasts) bovine growth serum (Cytiva, SH3054.03), 100× nonessential amino acids (Cytiva, SH30238.01), 200 μmol/L L-glutamine (Cytiva, SH30034.01), and 10 U/mL of penicillin–streptomycin (Cytiva, SV30010). Cell lines were passaged every 2 to 3 days. Primary fibroblasts were passaged when they became ∼80% confluent. Experiments were performed following 2 to 5 passages after thawing cells.

### Cell medium fractionation

To prepare large amounts of serum-free conditioned medium for fractionation from DiFi cells, hollow fiber bioreactors were used as previously described (32). Reactors were loaded with 1 to 5 × 10^8^ DiFi cells. A glucose utilization of 1 to 2 mg of glucose per day was used to confirm that cells had reached the equilibrium growth phase. Medium was harvested and processed as previously described (32, 33). To remove cellular debris, 20 mL of conditioned medium was centrifuged at 250 × *g*, then at 2,500 × *g* for 10 minutes following each harvest, and was gravity-filtered through a Millex 0.22-μm pore syringe filter (MilliporeSigma). Each pool was divided into 60-mL aliquots and stored at 4°C prior to fractionation. Medium fractionation into extracellular vesicle (EV) pellets, exomeres, and supermeres was performed by ultracentrifugation to permit quantification of DDR1 in different fractions as previously described (34). Fast protein liquid chromatography–size-exclusion chromatography (FPLC-SEC) was used to prepare supermeres for treatment experiments as previously described (34). We have previously shown that supermeres isolated by either ultracentrifugation or FPLC-SEC contain similar levels of DDR1 cECD (34).

### DDR1knockout in DiFi cells

To knock out *DDR1* in DiFi cells, lentiviruses containing puromycin resistance, GFP, Cas9 and DDR1 knockout (KO), and nontargeting control single-guide RNA (sgRNA) sequences were purchased from MilliporeSigma (human DDR1 KO sgRNA sequence: GTA​ACG​CAG​CCG​GTA​GCT​C). To transduce cells, 25 μL of virus was added to 15 mL of medium with 5% FBS supplemented with 1.2 μg/mL of polybrene and used to treat parental DiFi cells. After resting for 5 days, DiFi cells were treated with increasing concentrations of puromycin. To generate DDR1 KO and DDR1-expressing control clones, cells were FACS-sorted using BD Biosciences Aria III on GFP expression. Successful KO of DDR1 was validated in the clones by immunoblotting and tracking of indels by decomposition (TIDE) assay.

### TIDE assay

To validate DDR1 KO at the genomic level, genomic DNA from DDR1-expressing control and DDR1 KO DiFi clones was isolated using a Qiagen DNeasy Blood & Tissue kit (69506). Sequences surrounding the CRISPR target site were amplified by PCR (primer sequences: GCTCCTGGGACCTCTACTTC and CAGGGGAGGAGCAGGTTTAG). Amplicons were isolated by Qiagen PCR Purification kit (28104) and submitted to GenHunter for Sanger sequencing using the same primers. For each clone, three sequencing runs were performed. Sequence chromatograms were uploaded to https://tide.nki.nl/ to quantify DDR1 KO efficiency and mixture of KO alleles.

### Cell viability by CellTiter-Glo

To test DiFi viability in 3D culture, 500 DiFi cells were suspended in 5-μL collagen I/Matrigel domes (described in the “Three-dimensional colorectal cancer spheroid/fibroblast coculture” section) in white-walled 96-well plates (Greiner Bio-One, 655098). At the time of measurement, 100 μL of CellTiter-Glo reagent (Promega, G7572) was added to each well. Plates were rocked for 10 minutes, and the luminescence was read using a Synergy H1 plate reader. Three biological replicates were performed, each consisting of eight technical replicates.

### Three-dimensional colorectal cancer spheroid/fibroblast coculture

The spheroid/fibroblast coculture method was based on a method previously described by Strating and colleagues (35) with the following modifications. DiFi cells were trypsinized, syringed 5× with an 18-gauge needle, and counted using a Bio-Rad TC20 Automated Cell Counter. Cells were resuspended in a mixture of 20% Matrigel (Corning, 354234), 1.25 mg/mL of collagen I (Corning, 354249), 5× neutralization buffer [0.1 mol/L 4-(2-hydroxyethyl)-1-piperazineethanesulfonic acid and 2% NaHCO_3_ in minimal essential media α (Gibco, 12561-056)], and serum-free medium (see “Two-dimensional cell culture” methods). Three-dimensional culture gel composition was optimized by testing a range of collagen I and Matrigel concentrations. The gel composition used permitted optimal fibroblast adhesion to the dome surface and gel contractility. Domes containing 2,000 DiFi cells in 35 μL of gel were plated on 24-well glass bottom plates (MatTek, P24G-0-13-F). Pilot experiments found that the initial collagen structure was extraordinarily sensitive to the temperature of polymerization, which has been previously reported (36). When domes were transferred to the incubator immediately following plating, the domes that had been plated first exhibited distinct collagen organization to those that had been plated last. To account for this, domes were allowed to polymerize at room temperature for 1 hour prior to the addition of room-temperature medium and transferred to the cell culture incubator. DiFi cells were allowed to grow for 13 days, until spheroids had formed. Prior to the addition to cocultures in which collagen alignment was quantified, fibroblasts were stained with 1:200 CellBrite red (Biotium, 30023) for 20 minutes in suspension at 37°C, washed, and counted. In experiments comparing DDR1-expressing control and DDR1 KO DiFi cells, 12,000 fibroblasts were added directly to each well. In experiments in which supermeres were added, supermeres were first diluted in medium at 0.2 mg/mL and sterile filtered with a 0.22-μm syringe filter. Diluted supermeres were used to resuspend fibroblasts prior to the addition to the spheroids. Following fibroblast addition, cocultures were allowed to grow for 6 days prior to second harmonic generation (2HG) imaging. In T-cell invasion assays, domes were transferred to a new plate containing fresh medium 7 days following fibroblast addition to reduce the effects of factors accumulated in the medium on invasion. CD4^+^ or CD8^+^ T cells were sorted as described in the “Cells” section and were added immediately following transfer of the domes to new wells. In experiments lacking fibroblasts, spheroids were allowed to grow for 20 days. In experiments lacking DiFi cells, fibroblasts were added directly to the domes following 1-hour polymerization.

### Contraction assay

To measure the contractility of fibroblasts, 300 fibroblasts were suspended in 5-μL collagen I/Matrigel domes plated on 96-well low-adhesion plates (Falcon, 351172) and allowed to set for 1 hour at room temperature prior to the addition of room-temperature medium. The number of fibroblasts per dome was selected such that they resulted in an intermediate amount of contraction in the absence of supermere treatment. An optimal supermere concentration was determined by treating the HNPCC cell line with a range of 0 to 0.2 mg/mL supermeres (Supplementary Fig. S6). As it produced the largest effect, a concentration of 0.2 mg/mL supermeres was selected for all experiments. PRTH101, originally developed by Incendia Therapeutics, was synthesized. In experiments in which supermeres were pre-incubated with PRTH101 or an isotype control antibody (Bio X Cell, BE0297), supermeres were first diluted to 0.2 mg/mL in medium and sterile filtered, and 10 μg/mL of antibody was added. Supermere–antibody mixtures were incubated on a rocker at 37°C for 1 hour prior to the addition to the fibroblast cultures. To ensure that dome adhesion to the plate did not inhibit contraction, domes were gently dislodged from the plate with a pipette tip. Wells were imaged using MuviCyte at 3-hour intervals following plating for 36 hours. Dome diameters were measured in ImageJ (RRID: SCR_003070). Domes that were irregularly shaped at the first time point or were positioned in such a way that diameter measurement was impossible were excluded from analysis. Due to the objective nature of diameter measurement, the individual performing the analysis was not blinded to the condition. *N* = 3 to 10 technical replicates per condition were used. For biological replicates, two fibroblast cell lines (HNPCC and HCF) and three patient-derived primary fibroblasts (patients 6–8; see Supplementary Table S2 for patient information) were used. Primary fibroblasts were selected to ensure that both sexes were represented.

### 2HG imaging of collagen in 3D cultures

To image the collagen in 3D cultures, gels were fixed in 4% paraformaldehyde (PFA) PBS for 10 minutes and washed 2× in PBS. Domes were removed from plates and attached to the bottom of a 10-cm cell culture dish using a cyanoacrylate-based adhesive and submerged in PBS. Images were collected using a Nikon AX multiphoton microscope with 880-nm excitation using a 25× dipping objective. Signal was collected in the 4′,6-diamidino-2-phenylindole (DAPI) channel at 440 nm. Z-stacks consisted of 600 to 650 sections which started at the dome surface and were separated by 0.3 μm. For each dome, four images were collected. The ImageJ OrientationJ plugin (RRID: SCR_014796) was used to quantify collagen coherence. The average alignment across all z-slices was quantified to capture the total amount of collagen alignment an invading T cell would encounter over a given distance. In each condition, three biological replicates, each of three technical replicates were used. As the image analysis was fully automated, the individual performing the analysis was not blinded to the conditions.

### T-cell invasion assay

Prior to T-cell addition, DiFi spheroids with and without fibroblasts were grown in 3D culture as described in the “Three-dimensional colorectal cancer spheroid/fibroblast coculture” section. T cells were added 20 days after starting the culture. CD8^+^ and CD4^+^ T cells were stained with 1:200 CellBrite red (Biotium, 30023) for 20 minutes in suspension at 37°C, washed, and counted. Domes were transferred to fresh 24-well plates containing fresh medium. Fifty thousand CD8^+^ or CD4^+^ T cells were added to each well. After 24 hours, domes were transferred to a new plate and washed with PBS to remove cells that did not invade into the dome. Domes were fixed for 10 minutes in 4% PFA PBS and washed 2× in PBS. To quantify invasion of T cells, domes were visualized by epifluorescence and differential interference contrast (DIC) and the number of CellBrite-positive cells within each DiFi spheroid were manually counted by an unblinded individual. To ensure that only T cells that had successfully penetrated surrounding collagen to enter the dome were counted, analysis was limited to spheroids which were completely contained within the dome. Under each condition, three biological replicates, each of three technical replicates, were used.

### Human formalin-fix, paraffin-embedded samples

The formalin-fix, paraffin-embedded (FFPE) tissue microarray (TMA) consisting of 1-mm cores from 71 patients with MSS colorectal cancer tumors and five primary human FFPE colorectal cancer samples used for CASTA with accompanying histologic reports were obtained through the Collaborative Human Tissue Network (CHTN; IRB #101531; see Supplementary Tables S1 and S2). Samples were used in accordance with the VUMC IRBs.

### Sex and age as biological variables

Based on reports of collagen organization being age and sex specific (37), samples from patients from a range of ages and both sexes were chosen for the colorectal cancer TMA, CASTA analysis, and derivation of normal fibroblasts (see Supplementary Tables S1 and S2 for patient data.) Selection of samples for use in CASTA was limited to MSS tumors with a high ratio of stroma as determined by histology. The dataset allowed for the identification of genes associated with *DDR1* expression and collagen alignment. However, the use of only five samples does not allow for population-level conclusions to be drawn from these data.

### Multiplex immunofluorescence

Cyclic staining was performed on the colorectal cancer TMA using the GE Cell Dive as described previously (5). Antibodies used include pancytokeratin Alexa Fluor 488 primary conjugated (Santa Cruz Biotechnology, 53-9003-82), CD8 Alexa Fluor 647 primary conjugated (BioLegend, 13983), and CD4 Alexa Fluor 647 primary conjugated (Abcam, ab133616). To stain for DDR1, the D1G6 monoclonal antibody (mAb; Cell Signaling Technology, 5583) was directly conjugated to Alexa Fluor 555 by Cell Signaling Technology. Epithelial regions of the images were isolated by generating epithelial masks for 322 spots using ilastik trained on the PanCK channel. DDR1 immunoreactivity was quantified as mean fluorescence intensity in ImageJ (RRID: SCR_003070). CD8^+^ and CD4^+^ T cells were manually counted by an individual blinded to the DDR1 expression. Tumors were binned into DDR1 low and high, representing the bottom and top halves of the range of epithelial DDR1 immunoreactivity. The statistical significance of the differences in the CD8^+^ and CD4^+^ T-cell infiltration in DDR1-low and -high tumors was determined by Wilcox test for nonparametric data. A Student *t* test was used for parametric data.

### Immunoblotting

To assess DDR1 levels, cells were lysed at ∼80% confluency using Cell Signaling Technology lysis buffer (9803), spun for 6 minutes at 13,000 RPM in a tabletop centrifuge, and sonicated for 20 seconds at 4°C. Loading buffer was added, and samples were separated in a 9% SDS-PAGE gel, transferred to a nitrocellulose membrane, and probed using 1:1,000 Cell Signaling Technology DDR1 D1G6 mAb targeting the DDR1 C-terminus (5583) as described previously (18). In some experiments, serum-starved DiFi cells were treated with vehicle (20 mmol/L acetic acid) or 50 μg/mL of collagen I in 20 mmol/L acetic acid (Corning, 354249). After 24 hours, cell lysates were analyzed by immunoblotting for levels of phosphorylated DDR1 using 1:1,000 Cell Signaling Technology anti–DDR1 pY513 (14531S). Anti–β-actin 1:2,000 was used as a loading control (Sigma-Aldrich, A5318). To probe conditioned medium for DDR1 cECD, 10% trichloroacetic acid was added to conditioned medium for 30 minutes on ice to precipitate proteins. Samples were pelleted by centrifugation for 10 minutes at 10,000 × *g*, washed 2× with cold acetone prior to resuspension in lysis buffer, and loaded into SDS-PAGE gels. To detect DDR1 cECD in secreted fractions, no additional preprocessing of samples was performed prior to the addition of SDS-PAGE loading buffer. To detect DDR1 cECD, blots were blocked with Odyssey Intercept (TBS) Blocking Buffer (927-60001) and probed using 1:1,000 R&D anti–N-terminal DDR1 (AF2396). As a loading control, Ponceau S stains were performed (Sigma-Aldrich, P7170). All blots were probed using 1:5,000 LI-COR Odyssey secondary antibodies and imaged using an Odyssey XF imager.

### Enzyme-linked immunosorbent assay

To quantify DDR1 cECD in conditioned medium and secreted fractions by enzyme-linked immunosorbent assay (ELISA), Human DDR1 SimpleStep ELISA Kit (abcam, ab260063) was used according to the manufacturer’s instructions. DiFi-conditioned medium was diluted 1:64 before addition. To determine the relative amounts of DDR1 in secreted fractions, DiFi-conditioned medium was concentrated by centrifugation in a 100-kDa centrifuge filter (MilliporeSigma, UFC71008) and fractionated by ultracentrifugation as previously described. To fully account for all DDR1 in the conditioned medium, the flow-through of the concentration step and the supermere supernatant were also collected and analyzed by ELISA. The protein concentrations and volumes of each fraction were measured and used to calculate the amount of cell-free DDR1 present in each fraction as a percentage of the total secreted protein. To obtain results in the linear range, 0.122 mg of EVs, 0.234 mg of exomeres, and 0.125 μg of supermeres were added. Flow-through and the supermere supernatant were diluted 1:2. For each ELISA measurement, three technical replicates were performed.

### CASTA

An initial 30-μm section was cut by microtome from five human FFPE colorectal cancer tumor blocks followed by a serial 5-μm section. The 5-μm section was transferred to a spatial transcriptomics slide (10x Genomics, 100466) by CytAssist and was processed according to the standard Visium v2 protocol, which provided ∼50 μm resolution. RNA sequencing was performed using a NovaSeq X series with paired end reading targeting 125 million reads per sample. Reads were aligned using Space Ranger, and fiducial alignment was performed using Loupe Browser. To image the collagen, the 30-μm section was deparaffinized, rehydrated, and coverslipped in mounting medium consisting of 50% glycerol and 50% PBS. The section was imaged by 2HG on a Nikon AX MP with 880 nm excitation with a 25× objective. Z-sections were separated by 7 μm, and images were tiled across the section. Resulting images were stitched and z-projected. To determine the amount of alignment in each area, the OrientationJ ImageJ plugin was adapted using an in-house macro which quantified coherence in each region of the image. As collagen alignment occurred on larger scales than the resolution of the desired alignment heatmap, the macro assigned coherence values to pixels based on the surrounding 200 μm of the image. The heatmap and Visium data were registered using an in-house pipeline which leveraged the napari Python plugin (RRID: SCR_022765). To subset Visium spots into epithelium and stroma, Uniform Manifold Approximation and Projection for Dimension Reduction clusters were generated from the spots in each sample using the Seurat package in R (RRID: SCR_007322). The clusters were binned into epithelial, stromal, or ambiguous based on their overlap with the hematoxylin and eosin (H&E) image. For analyses comparing the gene expression and collagen alignment in the stroma to *DDR1* expression in the adjacent epithelium, a subset of stromal spots immediately adjacent to epithelial spots were used. To identify the genes correlated with alignment and *DDR1* expression in the adjacent epithelium, Spearman ρ and Bonferroni-adjusted *P* values were calculated for each gene using the SciPy package (RRID: SCR_008058) in Python.

### Plotting and statistics

All plots were made in R using the ggplot2 package (RRID: SCR_014601). Trendlines represent linear regression models. Statistics were performed using the rstatix (RRID: SCR_021240) and ggpubr (RRID: SCR_021139) packages. For comparison of two samples, Student *t* tests (for parametric data) and Wilcoxon tests (for nonparametric data) were performed. When comparing multiple samples, ANOVA was used followed by the Tukey *post hoc* test when comparing all samples and Dunnett test when comparing multiple samples with a control. *P* values < 0.05 were considered significant.

## Results

### DDR1 expression in MSS colorectal cancer is associated with reduced CD8^+^ T-cell infiltration

We previously reported that *DDR1* gene expression was correlated with decreased *CD8A* gene expression in MSS colorectal cancer (8). As DDR1 protein abundance is regulated at a posttranslational level (13, 38, 39), DDR1 mRNA and protein levels may not be well correlated. Thus, we performed immunofluorescence (IF) using CD8 and DDR1 antibodies to assess DDR1 immunostaining and CD8^+^ T-cell infiltration into the tumor proper of 71 human MSS colorectal cancer samples in a colorectal cancer TMA.

**Figure 1.**
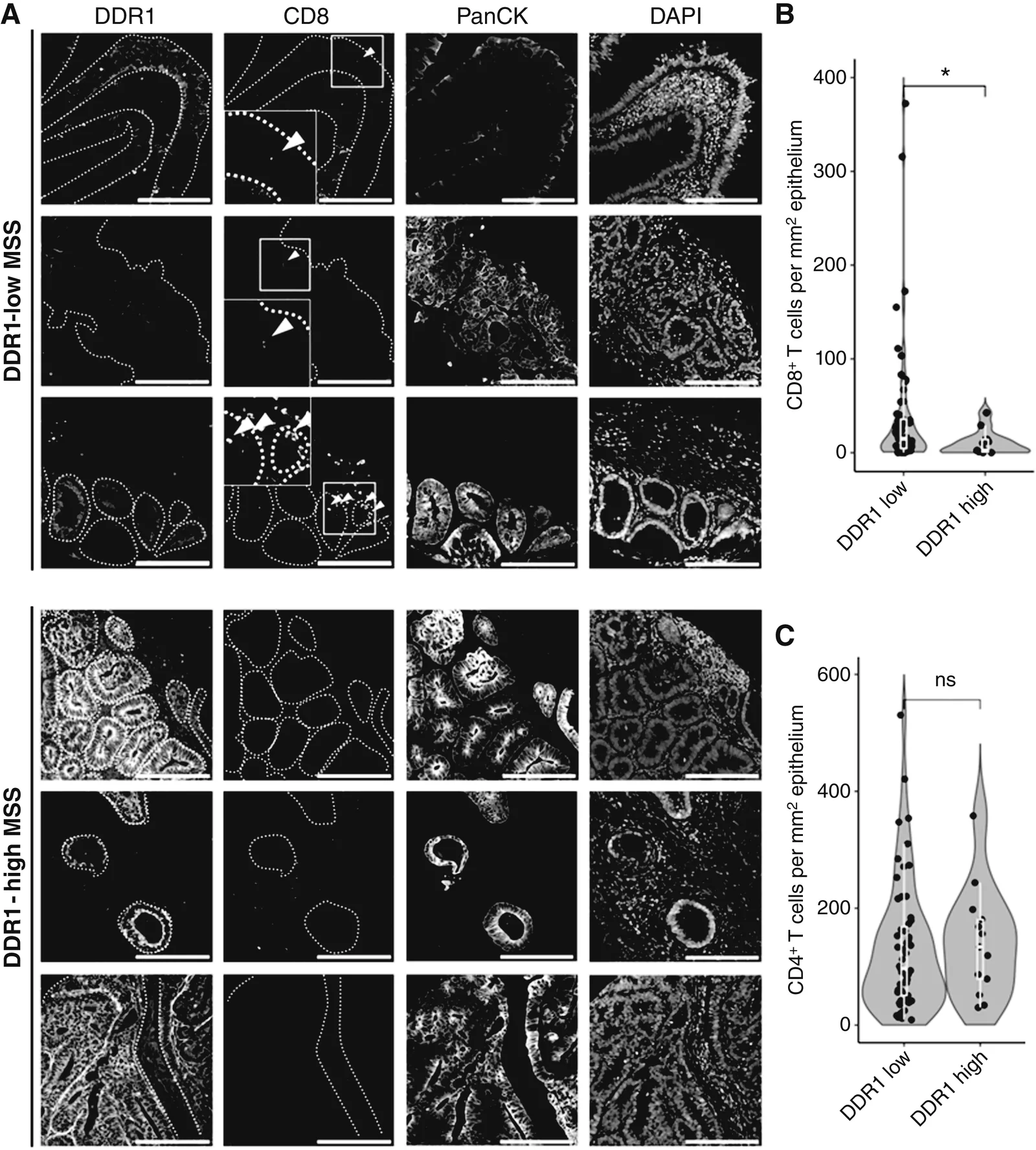
High DDR1 immunoreactivity is associated with decreased CD8^+^ T-cell infiltration in MSS colorectal cancer. **A,** Representative DDR1, CD8, PanCK, and DAPI immunofluorescence images of three 1-mm tumor cores from an MSS colorectal cancer TMA binned into DDR1 low and high based on epithelial DDR1 fluorescence intensity. Epithelial area outlined with dashed white line. Intraepithelial CD8^+^ T cells are marked by white arrowheads. Scale bars, 200 μm. **B** and **C,** Pirate plots of the relative number of CD8^+^ (**B**) and CD4^+^ (**C**) T cells per epithelial area in each DDR1 low and high MSS tumor sample. Each point represents the average of 1 tumor. *N* = 58 DDR1-low and 13 DDR1-high patients. ns, not statistically significant; *, *P* < 0.05.

Consistent with previous reports, DDR1 immunoreactivity was largely restricted to the epithelium (Fig. 1A; refs. 40, 41). CD8^+^ T-cell infiltration into the tumor epithelium is a prerequisite for effective ICB. As a result, the analysis needed to distinguish between intraepithelial and stromal CD8^+^ T cells. Therefore, we performed epithelial and stromal segmentation on the IF-stained TMA samples and analyzed only the epithelial segments, where most of the DDR1 was expressed.

DDR1-low MSS tumors had significantly higher numbers of CD8^+^ T cells per epithelial area than DDR1-high MSS tumors (Fig. 1A and B; Supplementary Fig. S1). Importantly, the relationship between T-cell infiltration and DDR1 was limited to CD8^+^ T cells, as CD4^+^ T-cell counts were unchanged between DDR1-low and -high MSS colorectal cancer samples (Fig. 1C; Supplementary Fig. S1).

### Identification of genes associated with collagen alignment and epithelial *DDR1* expression in human colorectal cancer samples

Having established the inverse relationship between DDR1 expression and intratumoral CD8^+^ T-cell infiltration in MSS colorectal cancer, we next aimed to identify genes, pathways, and cell types that could be involved in DDR1-mediated collagen alignment.

**Figure 2.**
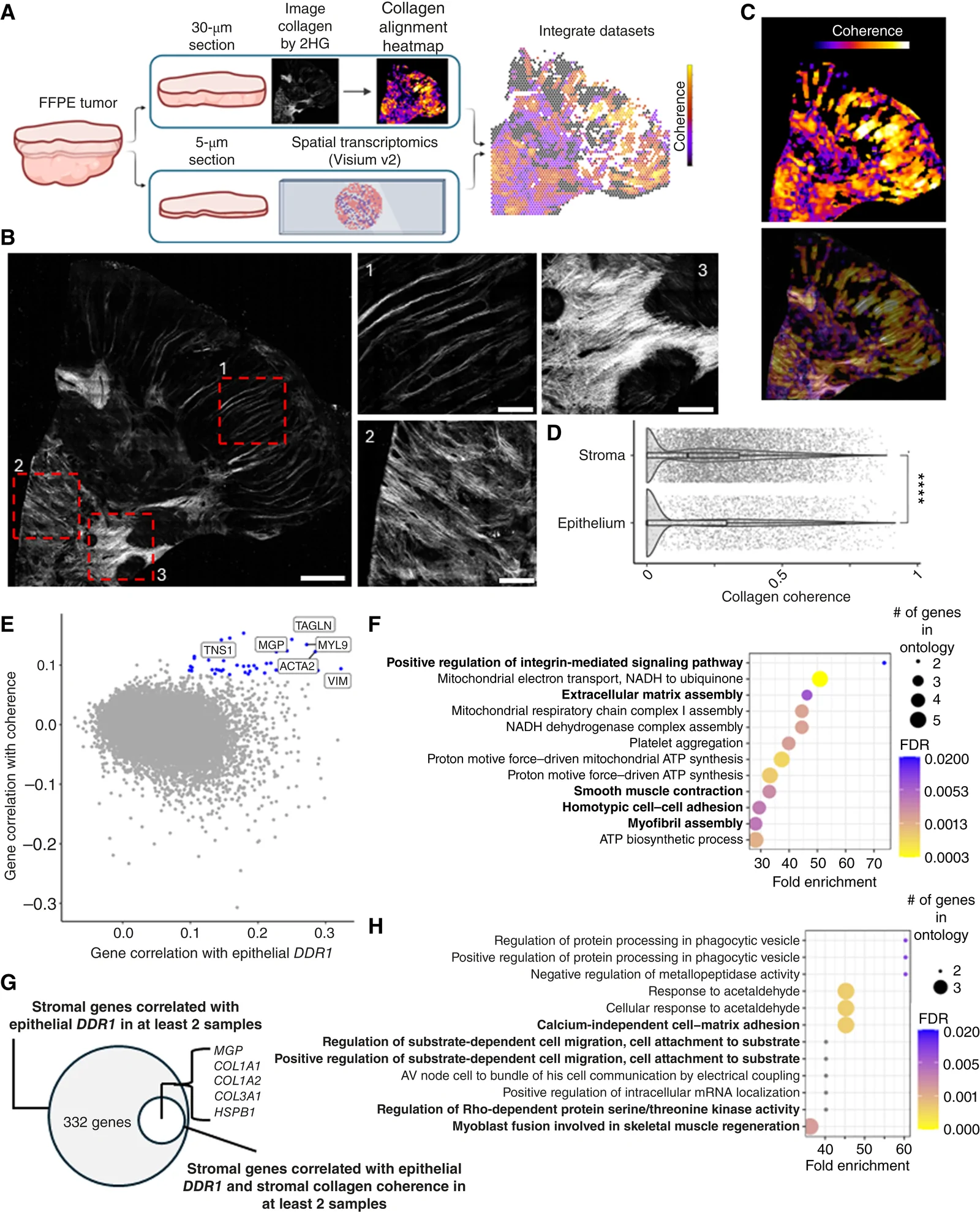
Stromal genes associated with *DDR1* and collagen alignment in colorectal cancer. **A,** Schematic of CASTA. **B,** Representative whole-section 2HG image of patient 1 tumor sample. Scale bars, 1 mm (left) and 400 μm (insets). **C,** Representative collagen coherence heatmap of patient 1 tumor sample. **D,** Pirate plot of collagen coherence in stromal and epithelial spots. Each point is one Visium spot. ****, *P* < 0.0001. **E,** Correlation of each gene with stromal collagen coherence and *DDR1* expression in adjacent epithelium. Positively correlated genes with an adjusted *P* < 0.05 highlighted in blue. **F,** Gene Ontology terms of genes identified in **E**. Contraction-related ontologies in bold. **G,** Overlap of stromal genes correlated with *DDR1* expression in two or more samples. **H,** Gene Ontology terms of stromal genes correlated with *DDR1* in two or more samples. Contraction-related ontologies in bold. Patient data in Supplementary Table S1.

As DDR1 was found to promote collagen alignment and in turn impair CD8^+^ T-cell infiltration into the tumor proper (22), we sought to determine whether epithelial *DDR1* expression in colorectal cancer is associated with increased stromal collagen alignment. To do this, we developed CASTA as a method to investigate the relationship between *DDR1* and collagen alignment in human colorectal cancer samples capable of capturing the spatial relationship between collagen alignment and gene expression (Fig. 2A). For this method, five human FFPE MSS colorectal cancer tumor blocks (see Supplementary Table S1 for clinical features of these five patients) were cut into serial sections with the first section being 30 μm and the subsequent section being 5 μm. The first section was imaged by 2HG (Fig. 2B). Spatial maps of collagen alignment were generated based on the measured coherence of the collagen in each region (Fig. 2C). The adjacent serial section was processed for standard Visium v2 spatial transcriptomics, which was then merged with the collagen alignment data (Fig. 2A). The resulting dataset integrated spatially resolved information about gene expression and collagen alignment in the tumors.

In the breast cancer mouse model described by Sun and colleagues (22), a barrier of aligned collagen in the stroma surrounds the tumor epithelium, hindering the access of CD8^+^ T cells. Consistent with the group’s findings, comparing collagen alignment in stromal and epithelial spots revealed that aligned collagen is indeed predominantly in the surrounding stroma rather than in the tumor epithelium itself (Fig. 2D).

Given that the DDR1 cECD is shed from DDR1-expressing tumor cells, we reasoned that if DDR1 cECD causes collagen alignment, the alignment would be highest in regions close to *DDR1*-expressing epithelium. A challenge in determining the correlation between *DDR1* expression and collagen alignment was how to correlate spatially separated epithelial *DDR1* expression and stromal collagen alignment. To overcome this challenge, the analysis was limited to stromal spots bordering the tumor epithelium and *DDR1* levels were quantified as the amount of *DDR1* expression in the nearest epithelial spot (Supplementary Fig. S2A).

We first used this analysis to confirm that *DDR1* expression in epithelial spots was associated with increased collagen alignment in the adjacent stromal spots by pooling the spots from all five patient samples (Supplementary Fig. S2B). We next generated a list of genes in stromal regions in all samples positively correlated with *DDR1* in the adjacent epithelium as well as collagen alignment (Fig. 2E). The genes identified were enriched for those expressed by fibroblasts and associated with cell contractility and cytoskeleton organization, such as smooth muscle α 2 actin and vimentin (Fig. 2E and F). We also generated a list of epithelial genes associated with *DDR1* expression and collagen alignment (Supplementary Fig. S3C). Although some genes were identified, no statistically significant gene ontologies were identified, suggesting that the association among *DDR1* expression, collagen alignment, and the expression of contraction-related genes was specific to the stroma.

Although pooling all five samples revealed which genes were associated with *DDR1* expression and collagen alignment across the population, an individual sample with strong correlations with a particular gene that is not correlated in other samples had the potential to confound the analysis. For example, we found that only patients 1 and 2 had an association between collagen alignment and platelet activation gene ontologies (Supplementary Fig. S3A). Therefore, we repeated the analysis on each sample individually (Supplementary Fig. S3A and S3B). We identified overlapping correlated genes and gene ontologies shared among the samples (Fig. 2G and H; Supplementary Fig. S3A and S3B). However, not all samples shared gene ontologies that correlated with *DDR1* expression or collagen alignment. This was primarily due to some samples having few total genes correlated with *DDR1* expression and collagen alignment, as well as variation in the ontologies identified. Although five samples are insufficient to draw broad conclusions about gene associations with *DDR1* expression and collagen alignment, ontologies associated with cell contractility were common to most samples (Supplementary Fig. S3A and S3B). To identify the most common ontologies associated with *DDR1* expression and collagen alignment, we focused our analysis on the shared ontologies. Gene ontologies of the *DDR1*-associated genes overlapping between at least two samples were enriched for genes associated with cell–matrix adhesion, regulation of cell contraction, and motility (Fig. 2H). Altogether, CASTA was able to capture the diverse gene programs associated with collagen alignment and *DDR1* expression in each sample. These results suggest that gene expression changes associated with collagen alignment and *DDR1* expression are predominantly composed of genes expressed by fibroblasts and involved in contractility, motility, and related processes.

### DDR1 KO in DiFi spheroid/fibroblast coculture resulted in decreased collagen alignment and CD8^+^ T-cell infiltration

**Figure 3.**
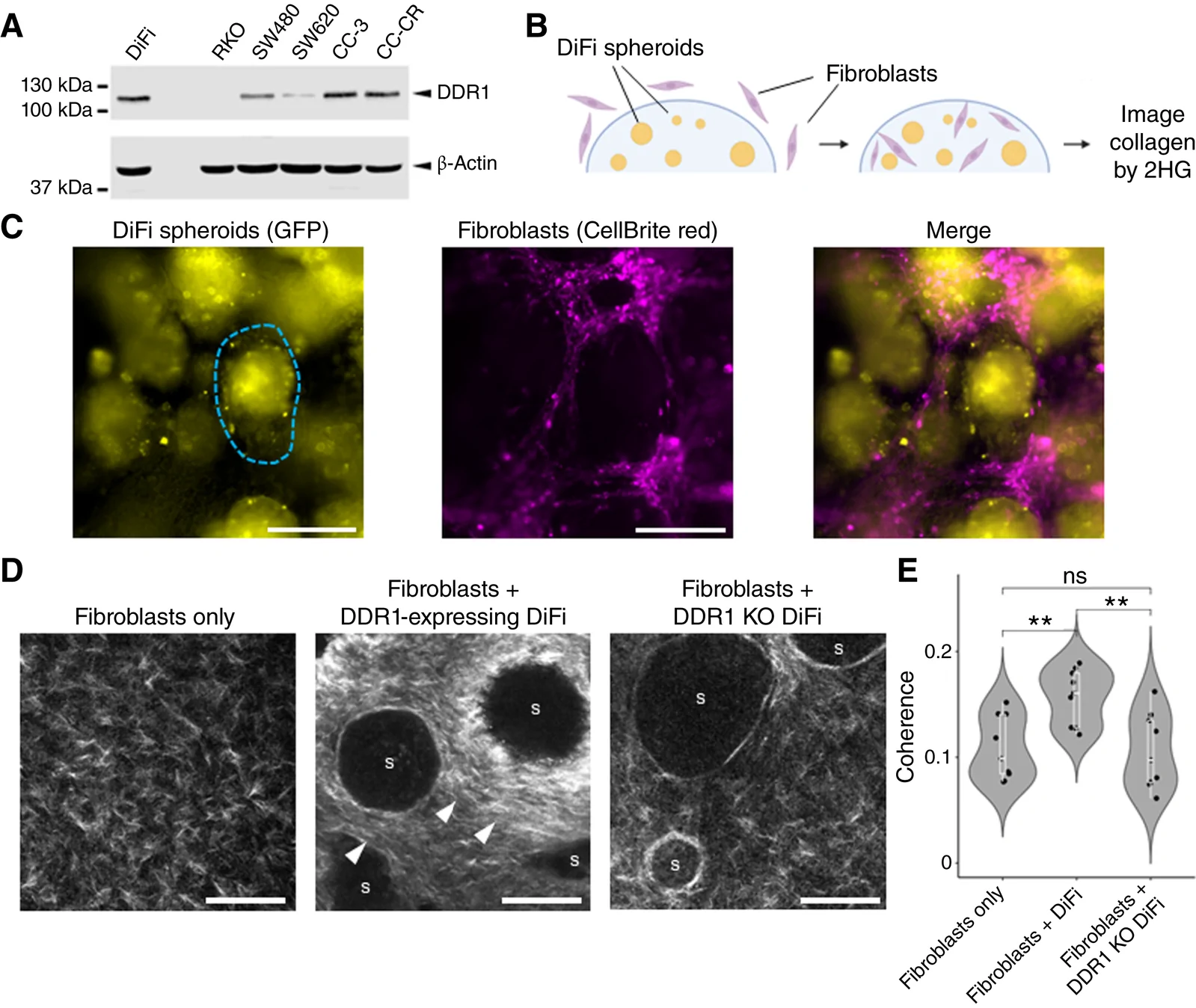
Coculturing DDR1-expressing DiFi spheroids with primary normal patient-derived fibroblasts promotes collagen alignment in 3D coculture. **A,** Immunoblot for DDR1 in colorectal cancer cell lines. **B,** Schematic of the DiFi spheroid/fibroblast coculture method. **C,** Representative epifluorescence images of DiFi spheroid (yellow, dotted cyan outline) and fibroblast (magenta) cocultures. Scale bars, 200 μm. **D** and **E,** Representative z-projections of 2HG images and pirate plots of collagen coherence in collagen/Matrigel domes containing fibroblasts and DDR1-expressing or DDR1 KO DiFi spheroids. White arrowheads point to regions of aligned collagen. White S denotes DiFi spheroids. Scale bars, 100 μm. Each point represents average coherence of a single dome. ns, not statistically significant; **, *P* < 0.01. *N* = 9 domes.

Based on the CASTA data that *DDR1* expression and collagen alignment were associated with fibroblast contraction, we developed a coculture system in which the effects of DDR1 expressed by human colorectal cancer cells on fibroblasts could be measured with respect to collagen alignment and T-cell infiltration. We evaluated DDR1 expression in human colorectal cancer cell lines by immunoblotting (Fig. 3A; Supplementary Fig. S4A) and selected DiFi cells for our coculture system as they express DDR1, are an MSS colorectal cancer cell line, and have a well-characterized secretome (26, 34). To determine if DDR1 is functional in DiFi cells, we treated the cells with collagen I and observed DDR1 autophosphorylation (Supplementary Fig. S4B).

We next generated DDR1 KO DiFi cells by CRISPR-Cas9. Elimination of DDR1 was validated by immunoblotting and TIDE assay (Supplementary Fig. S4C and S4D). As cell the growth rate could be a confounding variable, we measured cell viability of DDR1 KO and DDR1-expressing nontargeting control DiFi clones grown in a mixture of collagen I and Matrigel (see “Materials and Methods”). We identified DDR1 KO DiFi clone B as a clone that grew at a similar rate to the DDR1-expressing clone (Supplementary Fig. S4E), which was then used for subsequent experiments.

To measure the effects of DDR1 on collagen alignment and CD8^+^ T-cell infiltration in an *in vitro* model, we adapted a method first described for organoid/fibroblast coculture (35). DiFi spheroids were grown in a 3D dome of collagen I and Matrigel. Primary patient-derived colonic fibroblasts were added on top of the domes, which then infiltrated into the spaces between the spheroids (Fig. 3B). This method produced collagen I/Matrigel domes with pockets of DiFi cells surrounded by collagen permeated by fibroblasts, mimicking the organization of a tumor (Fig. 3C).

To test the impact of DDR1 on collagen alignment in the coculture system, we generated domes containing no spheroids, DDR1-expressing DiFi spheroids, or DDR1 KO DiFi spheroids. Primary normal human colonic fibroblasts were then added to the culture. We found that DiFi/fibroblast 3D cocultures containing DDR1-expressing DiFi spheroids aligned collagen more effectively than domes containing DDR1 KO spheroids or no spheroids at all (Fig. 3D and E). To confirm that fibroblasts were essential to observe the increase in collagen alignment, we repeated the experiment without adding fibroblasts, finding that collagen alignment was not promoted by DDR1-expressing DiFi spheroids in the absence of fibroblasts (Supplementary Fig. S5A and S5B).

**Figure 4.**
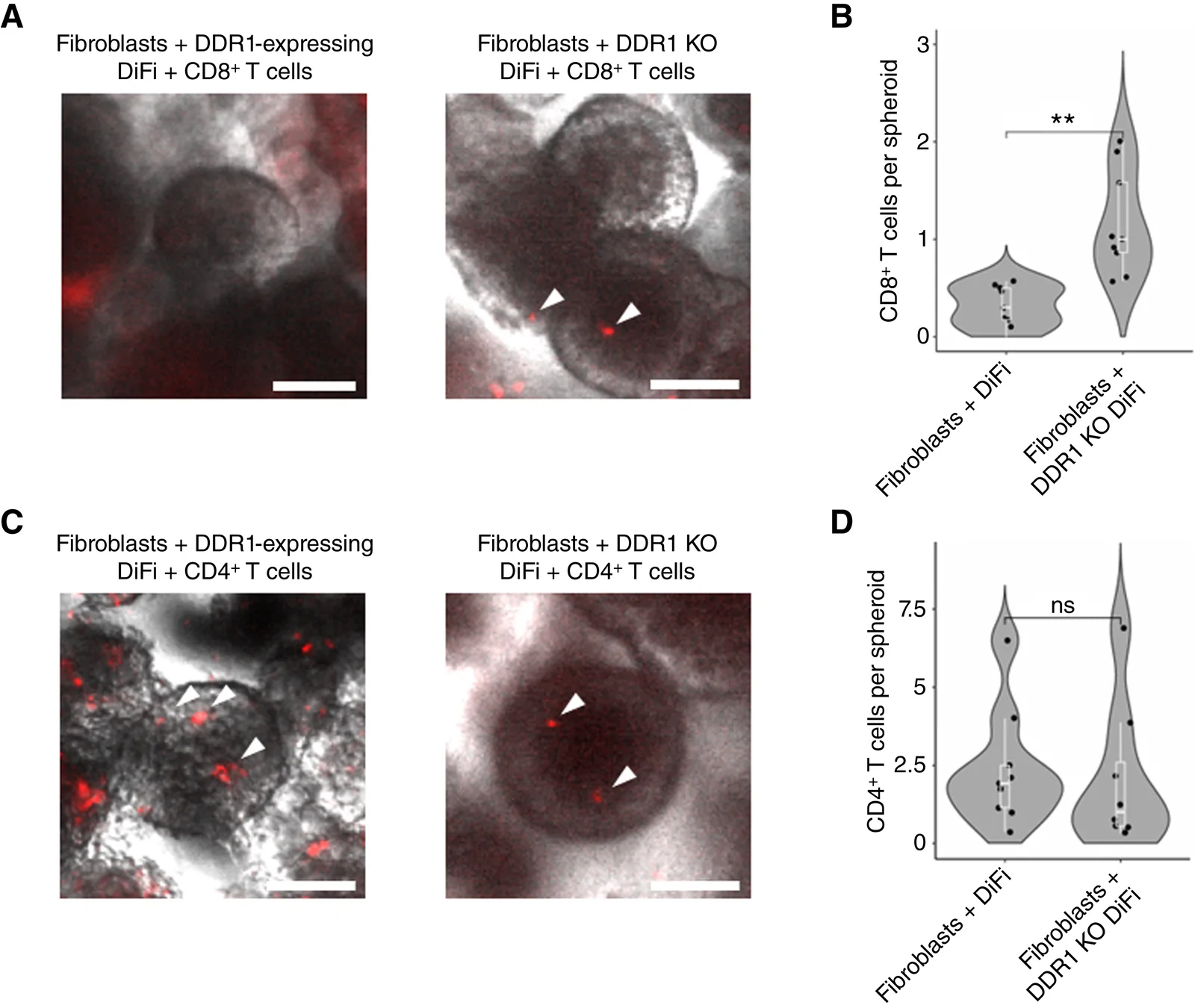
Coculturing DDR1-expressing DiFi spheroids with normal fibroblasts promotes CD8^+^ T-cell exclusion in 3D coculture. **A–D,** Representative merged DIC and epifluorescence images of CD8^+^ (**A** and **B**) and CD4^+^ (**C** and **D**) T cells (red, white arrowheads point to labeled T cells within spheroids) and pirate plots of average T-cell infiltration in DiFi spheroids after 24 hours. Scale bars, 25 μm. Each point is the average number of T cells per DiFi spheroid in a single dome. ns, not statistically significant; **, *P* < 0.01. *N* = 9 domes.

To test if DDR1 expression in colorectal cancer spheroids also resulted in CD8^+^ T-cell exclusion, CellBrite red–labeled CD8^+^ T cells were added to the cocultures 7 days following fibroblast addition. The number of infiltrating CD8^+^ T cells was significantly elevated in the DDR1 KO DiFi spheroids compared with DDR1-expressing DiFi spheroids (Fig. 4A and B). However, it was possible that DDR1 expression resulted in reduced CD8^+^ T-cell infiltration through a fibroblast-independent mechanism. We therefore repeated the experiment without adding fibroblasts and found that CD8^+^ T cells equally infiltrated DDR1 KO and DDR1-expressing DiFi spheroids (Supplementary Fig. S5C and S5D). Moreover, CD8^+^ T cells invaded approximately 10 times more effectively in the cocultures lacking fibroblasts, which is consistent for a role for fibroblasts in CD8^+^ T-cell exclusion independent of the effects of DDR1. Overall, these results suggest that DDR1-expressing DiFi spheroids and fibroblasts are able to promote collagen alignment and CD8^+^ T-cell exclusion in an *in vitro* 3D human colorectal cancer model.

As the multiplex IF (MxIF) analysis of human colorectal cancer samples revealed a correlation between DDR1 expression and CD8^+^, but not CD4^+^, T-cell infiltration into the tumor epithelium, we wondered if CD4^+^ T cells would infiltrate just as effectively into control and DDR1 KO DiFi spheroids in the 3D coculture system. We therefore repeated the T-cell infiltration experiments this time using CD4^+^ T cells. Consistent with the analysis of samples of patients with colorectal cancer, we found that DDR1 expression by DiFi cells had no impact on CD4^+^ T-cell infiltration, both with and without fibroblasts present (Fig. 4C and D; Supplementary Fig. S5E and S5F). These results suggest that the effects of DDR1-mediated T-cell exclusion are specific to CD8^+^ T cells.

### DDR1 cECD is released from colorectal cancer cells in supermeres

**Figure 5.**
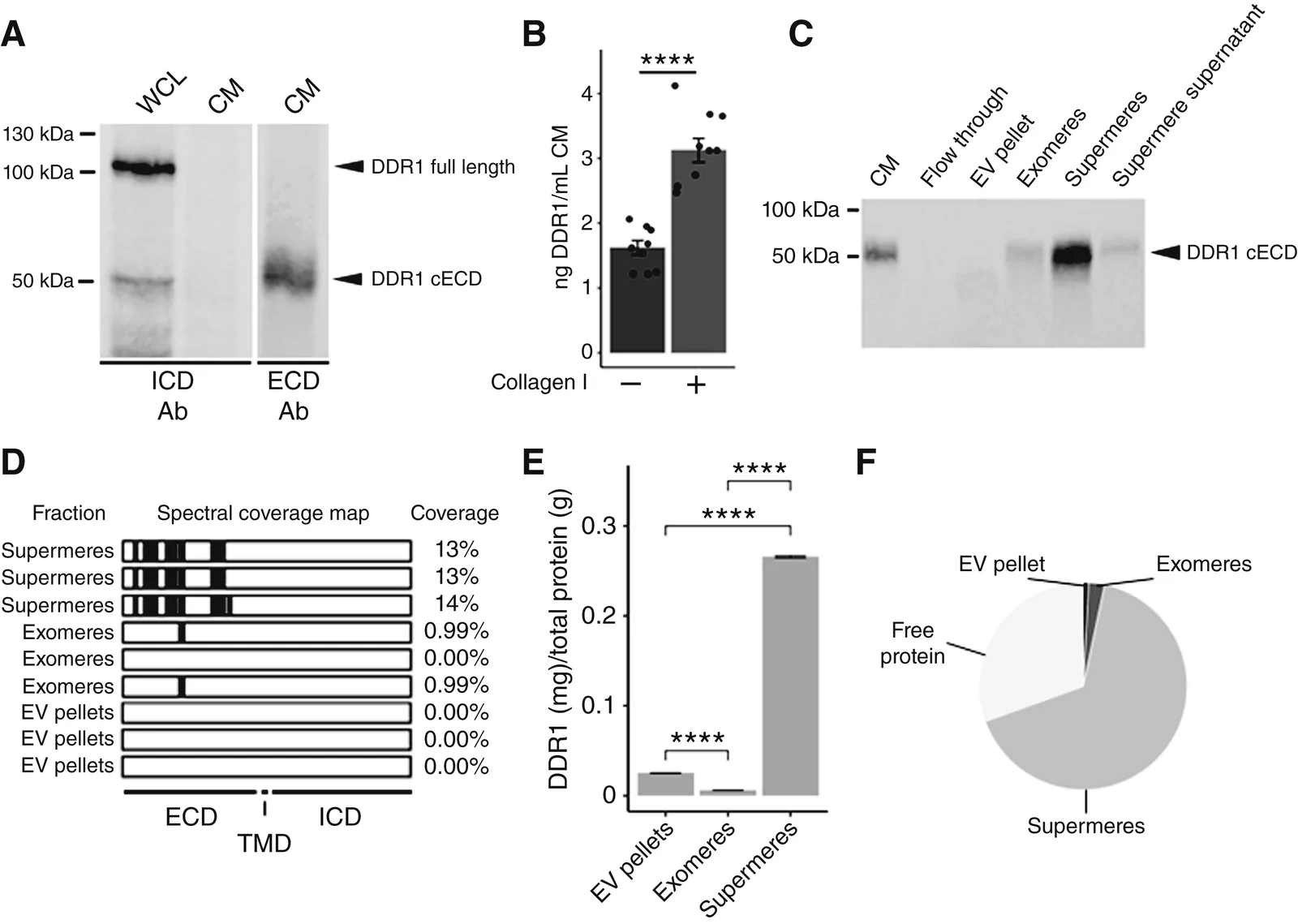
Collagen I–induced activation of DDR1 promotes release of supermeres containing DDR1 cECD from DiFi colorectal cancer cells. **A,** Immunoblots of DiFi whole-cell lysate (WCL) and conditioned medium (CM). Protein in DiFi CM was precipitated with the addition of 10% trichloroacetic acid. Immunoblots were probed with an intracellular domain (ICD; Cell Signaling Technology, 5583; left) and ECD (R&D, AF2396; right) antibody. Figure represents two separate blots. **B,** ELISA for DDR1 in CM from DiFi cells treated with collagen I (50 μg/mL) for 24 hours in 2D. Each data point is one measurement from three independent experiments performed in triplicate. Bar height is the measurement average ± SEM. ****, *P* < 0.0001. **C,** Immunoblots of secreted fractions of DiFi cells with a DDR1 ECD antibody (R&D, AF2396). **D,** Coverage and location of mapped DDR1 mass spectrometry counts from three replicates of EV pellets, exomeres, and supermeres from DiFi cells cultured in a 3D bioreactor (see “Materials and Methods”). Regions corresponding to DDR1 ECD, transmembrane domain (TMD), and ICD indicated. **E,** Representative levels of DDR1 in ultracentrifugation-fractionated DiFi cell CM as measured by ELISA. Experiment performed in triplicate. Values represent mean measurement ± SEM. ****, *P* < 0.0001. **F,** Relative amount of total secreted DDR1 in EVs (0.6%), exomeres (2.7%), supermeres (66.2%), and free protein (30.5%) as measured by ELISA from DiFi cells cultured in a 3D bioreactor. Representative experiment performed in triplicate.

We next determined if DDR1 cECD is released by DiFi cells and if the cECD was sufficient to increase collagen alignment. Immunoblots performed on cell lysates and conditioned medium using a DDR1 ECD–targeting antibody revealed the presence of DDR1 cECD only in conditioned medium (Fig. 5A). ELISAs, able to detect both full-length DDR1 and DDR1 cECD, showed that DDR1 levels were increased in conditioned medium following collagen I treatment (Fig. 5B).

We previously found that the growth-promoting activity of the EGFR ligand, amphiregulin (AREG), was significantly enhanced when AREG-containing EVs were presented to cells compared with equivalent amounts of recombinant protein (42). Thus, we investigated whether DDR1 cECD was released as free protein or in the form of a secreted nanoparticle such as EVs, exomeres (43), or supermeres (26, 34) and whether packaging into a nanoparticle may be required for induction of collagen alignment. DiFi cell–conditioned medium was fractionated into EVs, exomeres, and supermeres by FPLC-SEC (see “Materials and Methods”). Fractions from DiFi cells isolated using this method were extensively characterized and validated in a previous publication (34). DDR1 cECD levels were assessed by ELISA in each secreted fraction, revealing that the majority of DDR1 was found in supermeres (Fig. 5E and F). To ensure that the ELISA was able to detect both DDR1 cECD and full-length protein, immunoblots, performed on conditioned medium and individual fractions, revealed a band corresponding to the expected mass of DDR1 cECD in conditioned medium and supermeres (Fig. 5C). To further confirm that DDR1 cECD was present in supermeres, we analyzed our previously published proteomic data of DiFi EV pellets, exomeres, and supermeres (34) and found that DDR1 counts mapped only to the DDR1 ECD in supermeres (Fig. 5D).

### Fibroblasts treated with supermeres containing DDR1 cECD are more contractile than their DDR1 KO supermere–treated counterparts

**Figure 6.**
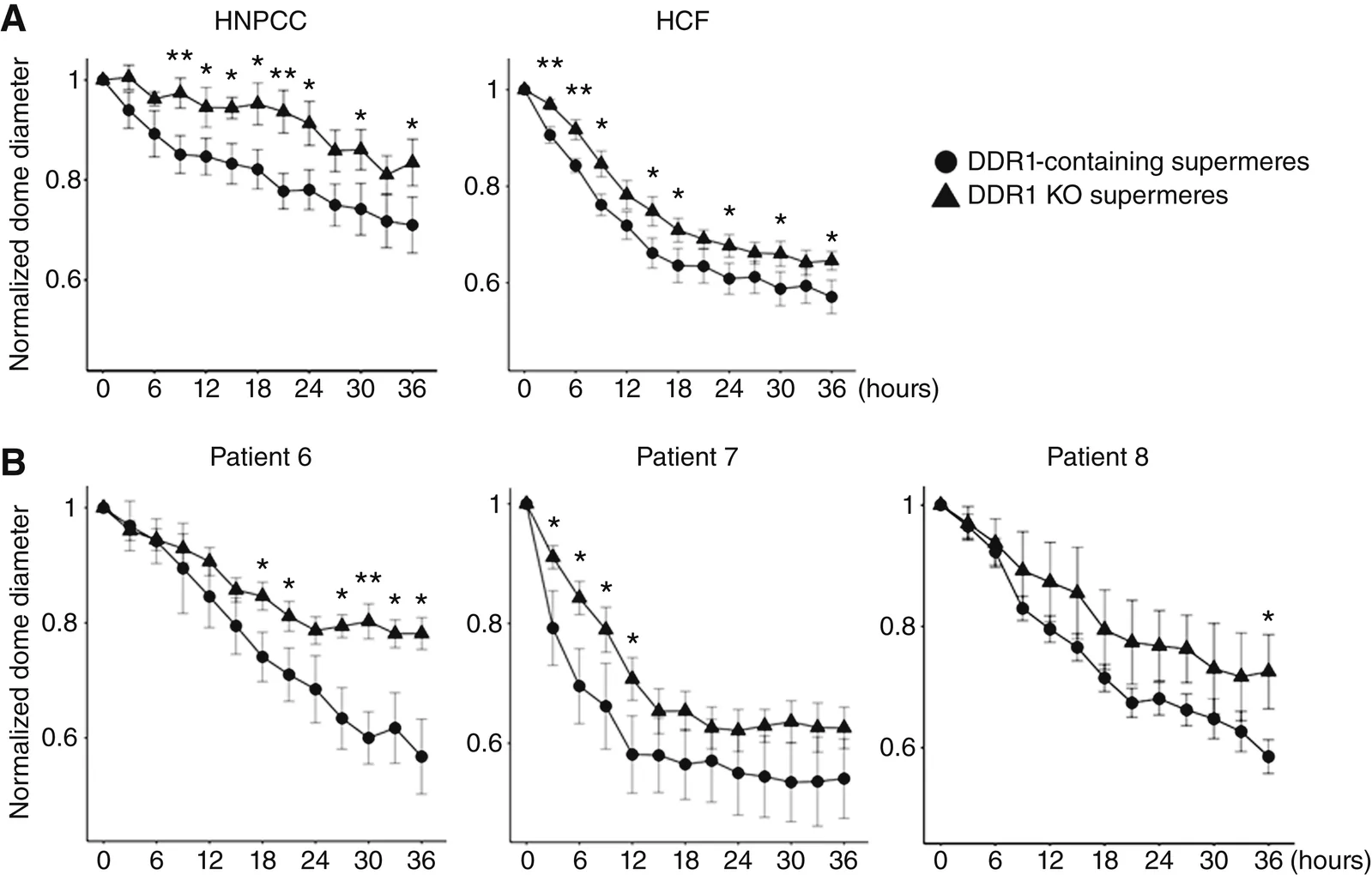
Supermeres containing DDR1 cECD promote contraction of normal fibroblasts. **A,** Diameters of collagen I/Matrigel domes containing HNPCC (left) and HCF (right) cell lines treated with supermeres derived from DDR1-expressing (circles) or DDR1 KO (triangles) DiFi cells. Data points represent the average diameter ± SEM. *, *P* < 0.05; **, *P* < 0.01. *N* = 5–9 domes. **B,** Diameters of collagen I/Matrigel domes containing primary fibroblasts isolated from the adjacent normal region of surgically resected colorectal cancer (patients 6 and 8) and adenoma (patient 7) treated with supermeres derived from DDR1-expressing or DDR1 KO DiFi cells. Diameters normalized to the first time point. Data points represent the average diameter ± SEM. *, *P* < 0.05; **, *P* < 0.01. *N* = 6–8 domes. Patient data in Supplementary Table S2.

CASTA revealed a spatial correlation between epithelial expression of *DDR1* and genes associated with fibroblast contraction in the nearby stroma (Fig. 2E–H; Supplementary Fig. S3A and S3B). Moreover, we previously showed that colorectal cancer–derived supermeres are capable of signaling between colorectal cancer cell lines (26). These findings led us to hypothesize that supermeres containing DDR1 cECD promoted fibroblast contraction, leading to collagen alignment. Thus, we treated primary normal fibroblasts from three patients (Supplementary Table S2) and two fibroblast lines (HNPCC and HCF) with DDR1 KO supermeres and supermeres containing DDR1 cECD in 3D collagen I/Matrigel domes and measured the diameter of the domes over time. For all the fibroblasts, supermeres containing DDR1 cECD promoted greater contraction compared with DDR1 KO DiFi supermeres (Fig. 6A and B). As discussed above, we previously found that AREG packaging into a secreted nanoparticle affects its signaling potency. We therefore wondered if free DDR1 ECD was sufficient to promote fibroblast contractility or if it must be associated with a supermere. To compare the effects of supermere-associated DDR1 cECD and free DDR1 ECD, we treated 3D fibroblast cultures with vehicle or recombinant DDR1 ECD. Results varied between patient samples, suggesting that only supermeres containing DDR1 cECD were consistently able to promote contraction (Supplementary Fig. S7A and S7B).

We next aimed to determine if reintroducing recombinant DDR1 ECD to DDR1 KO DiFi–derived supermeres was sufficient to promote contraction. Recombinant DDR1 ECD was premixed with DDR1 KO DiFi–derived supermeres and added to HNPCC and HCF cell lines and to the primary fibroblasts from patient 6. Adding mixed recombinant DDR1 ECD and DDR1 KO DiFi–derived supermeres to HCF and patient 6 fibroblasts did not promote contraction compared with the DDR1 KO DiFi–derived supermere–treated controls. In HNPCC fibroblasts, the introduction of recombinant DDR1 ECD resulted in slightly reduced contractility (Supplementary Fig. S8). These results suggest that induction of fibroblast contraction by DDR1 cECD–containing DiFi supermeres in previous experiments did not result from the simple combination of the DDR1 ECD and DiFi supermeres.

It has previously been proposed that DDR1 cECD–collagen binding is necessary to cause alignment (22). To test if supermere-associated DDR1 cECD–collagen binding was required for induction of fibroblast contractility, we used PRTH101, an mAb able to block DDR1 cECD–collagen binding (23). DDR1 cECD–containing supermeres were preincubated with PRTH101 or an isotype control and used to treat 3D cultures of HCF and HNPCC fibroblasts. Consistent with a model in which supermere-associated DDR1 cECD–collagen binding is dispensable for induction of contractility, neither fibroblast line exhibited decreased contractility when treated with supermeres preincubated with PRTH101 (Supplementary Fig. S9).

To investigate if fibroblast contractility correlated with collagen alignment, we added fibroblasts from patient 8 to 3D collagen I/Matrigel domes lacking DiFi spheroids and added vehicle, supermeres containing DDR1 cECD, DDR1 KO DiFi supermeres, or recombinant DDR1 ECD and measured the collagen alignment. Surprisingly, in this initial version of the experiment in which no DiFi cells were present, no changes in collagen alignment were observed, suggesting that colorectal cancer cells need to be present to observe the effects of the supermeres (Supplementary Fig. S7C and S7D). We therefore added vehicle, supermeres containing DDR1 cECD, DDR1 KO supermeres, or recombinant DDR1 ECD to 3D collagen I/Matrigel cocultures of primary fibroblasts from patient 8 and DDR1 KO DiFi spheroids. As expected, we found that treatment with recombinant DDR1 ECD resulted in significantly higher alignment than vehicle and that treatment with DDR1 KO supermeres resulted in a modest downward trend in collagen alignment compared with treatment with supermeres containing DDR1 cECD (Supplementary Fig. S7E and S7F). The results were consistent with the contraction results from the same fibroblast sample (Fig. 6B; Supplementary Fig. S7B). Thus, treatment of fibroblasts with supermeres containing DDR1 cECD caused more contraction than treatment with DDR1 KO supermeres, and the increased contraction was associated with increased collagen alignment.

## Discussion

**Figure 7.**
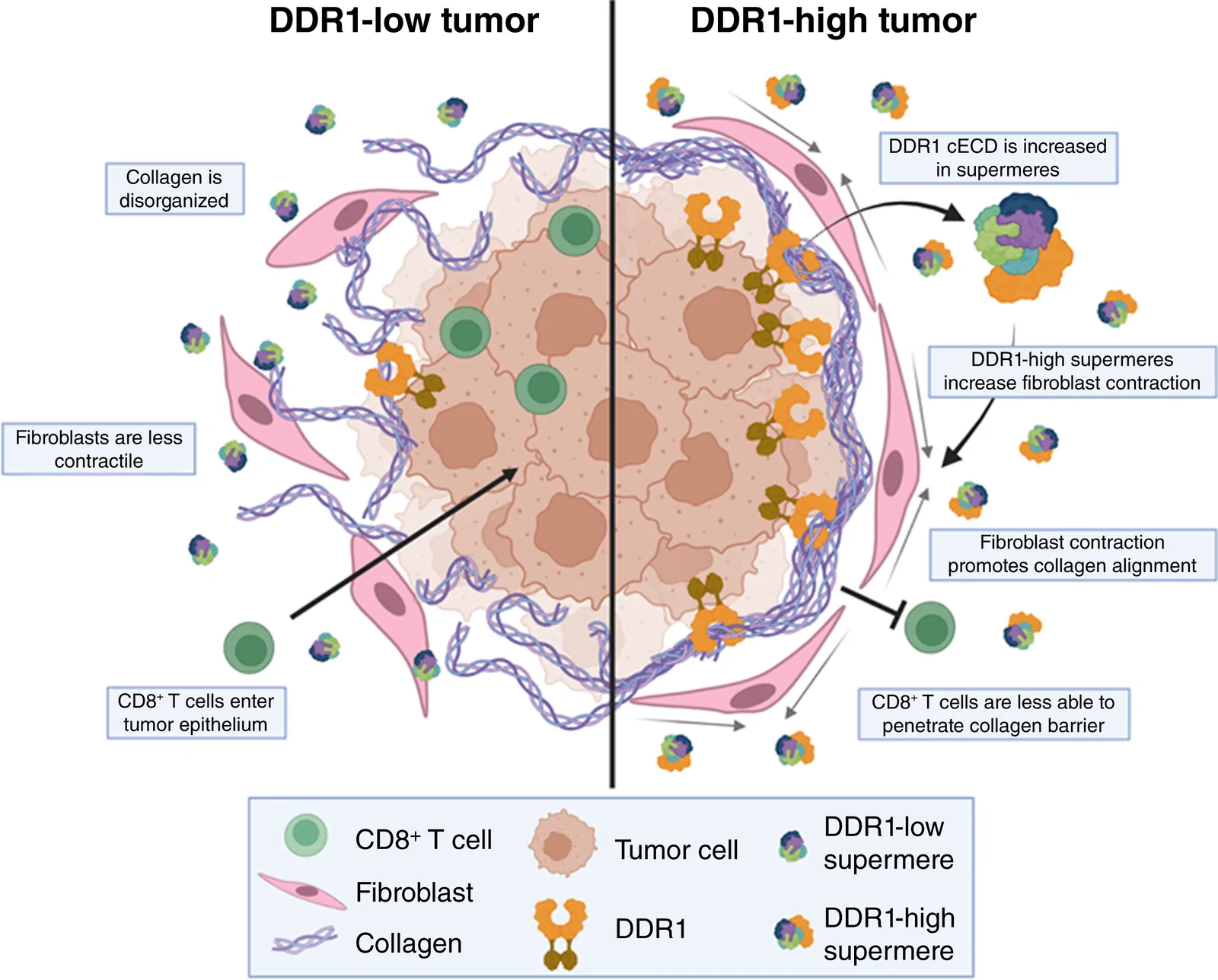
Model of the effects of colorectal cancer–derived supermeres containing DDR1 cECD on collagen alignment and CD8^+^ T-cell exclusion. In DDR1-low colorectal cancer, collagen is disorganized and CD8^+^ T cells are able to access the tumor epithelium (left). In DDR1-high colorectal cancer, collagen binding to DDR1 causes ECD cleavage, releasing it into the stroma in supermeres. Supermeres containing DDR1 cECD promote fibroblast contractility, resulting in collagen alignment, which impairs CD8^+^ T cells from entering the tumor epithelium (right). [Created in BioRender. Hamilton, M. (2026) https://BioRender.com/xw4jnrb.]

The goal of this study was to analyze the effects of DDR1 cECD on collagen alignment and CD8^+^ T-cell exclusion in MSS colorectal cancer. Based on the results, we propose a model in which DDR1 cECD is released from MSS colorectal cancer cells into the stroma packaged in supermeres where it promotes the contraction of fibroblasts, leading to collagen alignment and CD8^+^ T-cell exclusion (Fig. 7).

We found that DDR1 cECD was associated with colorectal cancer supermeres, an amembranous nanoparticle we recently discovered that is replete with biomarkers (26, 34). We previously identified the cECD of DDR1 in supermeres (34). This work expands on the role of supermeres by describing a novel functional role for supermeres in CD8^+^ T-cell exclusion in colorectal cancer. Although there is a pool of free DDR1 cECD that is not associated with supermeres (Fig. 5C and F), most experiments in which fibroblasts were treated with purified recombinant DDR1 ECD did not result in fibroblast contraction, highlighting the importance of supermeres containing DDR1 cECD in fibroblast dynamics. We propose that supermeres from colorectal cancer exert effects on stromal fibroblasts and that the presence of DDR1 cECD modulates their effect to increase fibroblast contraction. This model further points to a role for supermeres in regulation of cell contractility, which has broad implications in cancer and development (44). Future work should delineate the effects of RTK signaling of DDR1 from the effects of DDR1 cECD.

We found that normal fibroblasts promoted collagen alignment in the presence of DDR1-expressing DiFi spheroids. Critically, the effects of DDR1 expression on collagen alignment and CD8^+^ T-cell exclusion were only observed in cocultures that included fibroblasts, indicating that contractile cells are essential. Taken together, these results suggest that DDR1-expressing DiFi cells and fibroblasts are sufficient to induce collagen alignment in a 3D coculture model of colorectal cancer. The previously proposed model of DDR1 cECD–mediated immune exclusion depended on DDR1 binding to collagen to promote its alignment (22). Here, we refined the mechanism of DDR1-mediated collagen alignment. Our discovery that DDR1 did not promote collagen alignment when fibroblasts were absent and that pre-incubation of supermeres with PRTH101 did not reduce their induction of collagen alignment supports a model in which supermere-associated DDR1 cECD promotes collagen alignment through its effect on stromal fibroblasts, rather than through collagen binding.

CASTA represents a novel approach to analyze the relationship between gene expression and collagen alignment. Although others have characterized changes in extracellular matrix composition associated with collagen alignment (45), CASTA identifies the cell types and pathways associated with collagen alignment. CASTA is a powerful tool that could be used to characterize gene expression associated with collagen alignment in other settings, such as other cancer types, fibrosis, and in developmental processes involving collagen organization such as cartilage, muscle, and tendon.

We used CASTA to identify genes associated with collagen alignment and *DDR1* expression, which were enriched for fibroblast-expressed genes involved in contraction and related processes like cell–matrix adhesion and motility, consistent with existing models of contraction-mediated collagen alignment (46). Importantly, by performing CASTA on several samples, we found that genes associated with *DDR1* expression and collagen alignment were highly diverse. Although only a subset of genes was found to be associated with collagen alignment across multiple samples, we nevertheless found that the samples tended to share contraction-related gene ontologies. Moreover, treating different fibroblast samples with supermeres resulted in variable scales and timing of the effects of DDR1 on contraction. These findings suggest that the responses to supermeres containing DDR1 cECD are diverse but predominantly result in an increase in cell contractility, causing collagen alignment.

ICB has revolutionized cancer treatment. However, few patients with colorectal cancer have been able to benefit from its increased efficacy and decreased toxicity compared with chemotherapy. Our results support DDR1 as a tractable target to enable the use of ICB in the treatment of MSS colorectal cancer and provide valuable insights into how and why specific DDR1-targeting therapies may succeed or fail. Consistent with our model that DDR1 cECD, not its kinase domain, promotes immune exclusion, small molecules targeting DDR1 kinase activity have not found success as anticancer drugs (47). Additionally, DDR1 cECD-neutralizing mAbs, which improve the efficacy of ICB in mouse models, have been developed (22, 23, 48). The results here suggest that DDR1 binding to collagen is not essential for its promotion of immune exclusion. As a result, additional work is needed on how best to inhibit the effects of supermeres containing DDR1 cECD on fibroblasts.

Our study demonstrates that DDR1 immunoreactivity correlates with reduced CD8^+^ T-cell infiltration into MSS tumor epithelium, which is consistent with a role for DDR1 in CD8^+^ T-cell exclusion in colorectal cancer. However, CD4^+^ T-cell infiltration does not correlate with DDR1 at the protein level. In support of this finding, DDR1 expression did not affect CD4^+^ T-cell infiltration of 3D cocultures of DiFi spheroids and fibroblasts. A possible reason for the difference between CD8^+^ and CD4^+^ T-cell exclusion is their differential sensitivity to LAIR1-mediated exhaustion in dense networks of cross-linked collagen, which has been explored in a lung cancer model (49).

Despite the strong negative association between DDR1 immunoreactivity and CD8^+^ T-cell infiltration in the MSS TMA samples, we found that not all DDR1-low tumors had high CD8^+^ T-cell infiltration within the tumor proper. Therefore, DDR1 may be dispensable for intratumoral CD8^+^ T-cell exclusion in some tumors. This suggests that some patients with MSS colorectal cancer may respond to ICB (50). These results also suggest that some MSS tumors employ DDR1-independent mechanisms to exclude CD8^+^ T cells. Predictive markers will need to be established before attempting to employ DDR1 cECD–targeting therapies in conjunction with ICB in patients with MSS colorectal cancer to identify those most likely to respond. Although MSI-H colorectal cancers contain a greater number of mutations than MSS colorectal cancers, cancers with low mutational burdens also may have increased neoantigen presentation (6, 7). As an example, work is underway to target cryptic neoantigens in low-mutational cancers (6, 7). Additionally, we have recently found that increased CD8^+^ T-cell infiltration precedes hypermutation in MSI-H colorectal cancer precancerous lesions (5). As a result, targeting DDR1 in MSS colorectal cancer may be especially effective when coupled with cryptic neoantigen-targeting immunotherapies. Taken together, these results underscore the value of DDR1 as a target in MSS colorectal cancer treatment, as well as the need for the identification of biomarkers to stratify patients into those that are likely to respond to DDR1-targeted therapy.

## Supplementary Material

Supplementary Figure S1 Figure S1. DDR1 immunoreactivity is associated with decreased CD8+ but not CD4+ T-cell infiltration of CRC tumor epithelium.

Supplementary Figure S2 Figure S2. Epithelial DDR1 expression is associated with collagen alignment in the stroma.

Supplementary Figure S3 Figure S3. Identification of stromal genes associated with epithelial DDR1 expression and stromal collagen alignment in CRC.

Supplementary Figure S4 Figure S4. DDR1 expression in CRC cell lines.

Supplementary Figure S5 Figure S5. DDR1-expressing DiFi spheroids alone are not sufficient to promote collagen alignment and CD8+ T-cell exclusion in 3D cultures.

Supplementary Figure S6 Figure S6. Effects of a range of supermere concentrations on HNPCC contractility.

Supplementary Figure S7 Figure S7. Effect of recombinant DDR1 ECD on contraction of normal fibroblasts and collagen alignment.

Supplementary Figure S8 Figure S8. Reintroducing recombinant DDR1 ECD to DDR1 KO supermeres does not rescue fibroblast contractility.

Supplementary Figure S9 Figure S9. PRTH101 does not inhibit DDR1-expressing DiFi supermere induction of contraction in fibroblasts.

Supplementary Table S1 Table S1. Patient data for samples analyzed by CASTA.

Supplementary Table S2 Table S2. Patient data for primary colon fibroblasts.

## Data Availability

The transcriptomics data are publicly available in Gene Expression Omnibus (RRID: SCR_005012; GSE311294). Other data generated in this study are available in the article, Supplementary files, or upon request to the corresponding author.

## Code Availability

Code used for this project is available on GitHub (https://github.com/maxwellh-bit/CASTA).
